# Climate change risks pushing one-third of global food production outside the safe climatic space

**DOI:** 10.1016/j.oneear.2021.04.017

**Published:** 2021-05-21

**Authors:** Matti Kummu, Matias Heino, Maija Taka, Olli Varis, Daniel Viviroli

**Affiliations:** 1 Water and Development Research Group, Aalto University, Espoo, Finland; 2 Department of Geography, University of Zürich, Zürich, Switzerland

**Keywords:** crop production, livestock production, safe operating space, climatic conditions, climate change, Holdridge life zones

## Abstract

Food production on our planet is dominantly based on agricultural practices developed during stable Holocene climatic conditions. Although it is widely accepted that climate change perturbs these conditions, no systematic understanding exists on where and how the major risks for entering unprecedented conditions may occur. Here, we address this gap by introducing the concept of safe climatic space (SCS), which incorporates the decisive climatic factors of agricultural production: precipitation, temperature, and aridity. We show that a rapid and unhalted growth of greenhouse gas emissions (SSP5–8.5) could force 31% of the global food crop and 34% of livestock production beyond the SCS by 2081–2100. The most vulnerable areas are South and Southeast Asia and Africa's Sudano-Sahelian Zone, which have low resilience to cope with these changes. Our results underpin the importance of committing to a low-emissions scenario (SSP1–2.6), whereupon the extent of food production facing unprecedented conditions would be a fraction.

## Highlights

• Safe climatic space method to assess climatic niche for global food production
• Climate change risks pushing 1/3 of food production outside safe climatic space
• Following the Paris Agreement would reduce this risk considerably
• We call for interlinked climate change mitigation and adaptation actions

## Science for society

Food production systems developed under stable Holocene climate conditions. To identify these conditions for the first time, we developed the novel concept of safe climatic space. We show that unhalted growth of greenhouse gas emissions could force nearly one-third of global food crop production and over one-third of livestock production beyond this safe space by 2081–2100. The most vulnerable areas are South and Southeast Asia and Africa's Sudano-Sahelian Zone, where a high risk of leaving these safe climatic conditions is combined with low resilience. Our findings reinforce existing studies showing that if warming cannot be limited to 1.5–2°C, humanity will be forced into a new era in which past experience is of reduced validity and uncertainties increase dramatically. Future policies should concentrate on actions that simultaneously mitigate climate change and increase sustainably the resilience of food systems and societies.

## Graphical abstract

**Figure:**
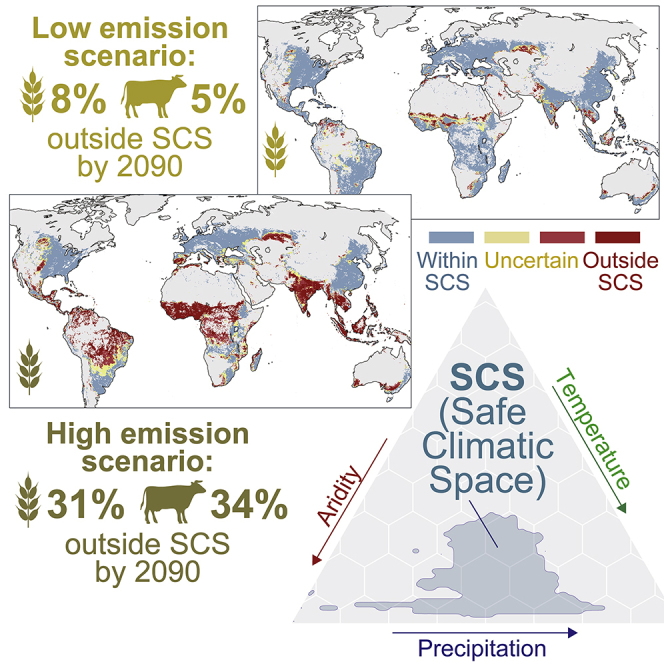

## Teaser

We developed the novel concept of safe climatic space, which allows us, for the first time, to identify the climatic conditions under which food production developed during the stable Holocene climate conditions. We show that nearly one-third of global food crop production and over one-third of livestock production could be forced beyond these suitable conditions by 2081–2100 if we cannot limit the warming to 1.5–2°C. The most vulnerable areas would be South and Southeast Asia and Africa's Sudano-Sahelian Zone.

## Introduction

Ecosystems and human societies have adapted to relatively stable Holocene climate conditions over several millennia.^1^^,^^2^ The majority of food production is based on agricultural practices developed for these conditions.^2^^,^^3^ There are already signs that the recent, accelerating global environmental change is affecting many important crops throughout the planet.^4^^,^^5^ Often the change is manifested in several indicators. This also applies to climate change, projected to change temperature and rainfall patterns, as well as aridity arising from these.^6^ These key parameters directly affect societies and their life-sustaining activities such as food production^7^^,^^8^ and maintaining water availability.^9^

Various studies have assessed the changes in agricultural conditions under climate change^10^, ^11^, ^12^ by analyzing the changes in climatic conditions^12^, ^13^, ^14^ and their potential impact on yields.^11^^,^^15^^,^^16^ It would, however, be important to also understand which areas might experience a truly novel climate under which no major agriculture exists today, along the lines of safe operating space (SOS) and climate niche concepts for human societies.^17^ SOS by definition^2^ refers to the Earth system conditions that would sustain human life as we know it. Although the planetary boundary framework includes an SOS for climate change,^18^ it is defined through global atmospheric carbon dioxide concentration and does not specify climatic thresholds that could be applied on a local scale. Xu et al.,^17^ in turn, argue that it is necessary to “understand climatic conditions for human thriving,” as it might be difficult to adapt to new climatic conditions at the pace projected by climate change. They find that a considerable part of the population will fall outside the temperature niche due to climate change.

**Figure 1:**
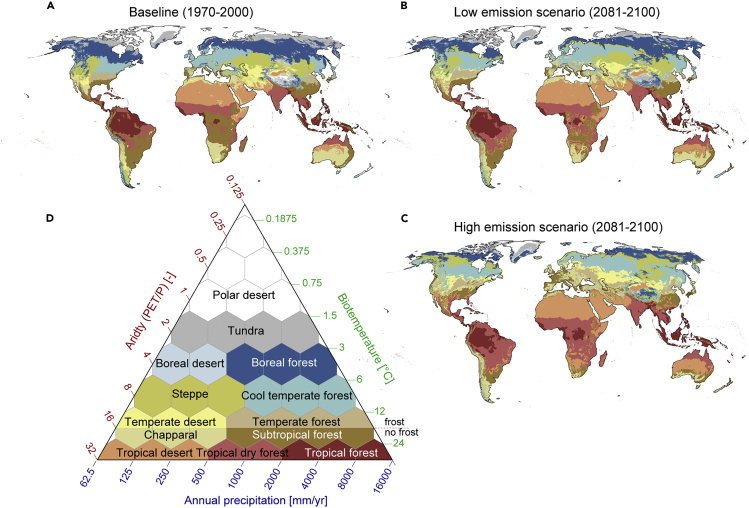
Maps and definition of Holdridge life zones (A–D) HLZ maps are shown for the baseline period (1970–2000) (A) as well as two climate change scenarios for 2081–2100 (B and C). Low emission scenario refers to the SSP1–2.6 scenario, while high emission scenario refers to the SSP5–8.5 scenario under the CMIP6 framework. The Holdridge triangle (D) shows the location of each HLZ in relation to biotemperature, potential evapotranspiration ratio, and annual precipitation; here the original 38 zones were aggregated into 13 zones following Leemans^30^ (experimental procedures). The maps (A–C) illustrate the same color classes as the triangle (D). The Holdridge triangle (D) is modified from Halasz.^31^ Note: Antarctica was part of the analysis but is not shown in the maps. Data for the Holdridge zones, as for all four assessed time periods (see experimental procedures), are available at the link provided in the data availability statement. “PET” stands for potential evapotranspiration and “P” stands for precipitation.

Changes in multiple climatic characteristics can be simultaneously measured with, for example, climate classifications such as the Holdridge life zone (HLZ) concept^19^^,^^20^ or the Köppen-Geiger climate classification.^21^ As the Holdridge concept is not limited to mapping the categorical changes, but also allows us to assess the magnitude and direction of changes, it is a more appropriate method for assessing the magnitude and direction of potential future changes in climatic conditions across the globe. The HLZ concept divides the Earth into 38 zones based on three climatic factors: annual precipitation, biotemperature, and aridity (Figures 1 and S1). It also considers whether an area experiences frost.^19^ All these factors are important for agriculture, both livestock^17^^,^^22^^,^^23^ and crop production.^24^ Previously, the HLZ concept has been successfully used for biomass estimations,^25^ as well as for analyzing climate-soil^26^ and climate-vegetation^27^ relationships, among other fields. Although studies mapping HLZs under future climates exist, these are conducted either at a regional scale^28^^,^^29^ or with simplistic climate scenarios (double CO_2_ emission).^30^ Thus, no up-to-date future scenarios for HLZs exist.

In this study we aim to go beyond the existing studies by first defining the novel concept safe climatic space (SCS) by using a combination of three climatic parameters in an integrated way, instead of assessing a single indicator at the time. The use of the HLZ concept allows us to do this. SCS is defined here as the climate conditions to which current food production systems (here crop production and livestock production, separately) are accustomed (experimental procedures; Figure S3), an analog to SOS concepts such as planetary boundaries^2^^,^^18^ and climatic niche.^17^ Our suggested SCS framework using Holdridge zoning provides thus a novel concept to define the climatic niche for current food production and allows us to holistically study the multifaceted and spatially heterogeneous risks of climate change on it. To assess these risks, we link the climate-change-induced alterations to HLZs over the coming 80 years with spatial gridded global datasets of (1) current production of 27 major food crops^32^ (experimental procedures) and (2) current livestock production of seven major livestock types,^33^ as well as (3) the resilience of human societies to cope with these changes.^34^ We find that a rapid and unhalted growth of greenhouse gas (GHG) emissions (SSP5–8.5 climate change scenario; “SSP” stands for shared socioeconomic pathways) could force one-third of global food production beyond the SCS by 2081–2100. The data for the current situation (year 2010) allow us to identify the current food production areas in which an elevated risk of leaving the SCS coincides with low capacity of the society to cope with additional stresses.

## Results

### Largest changes in polar regions, mountains, and the Sahel

We estimated the HLZs for baseline conditions (1970–2000) as well as for future conditions (2021–2040, 2041–2060, 2061–2080, and 2081–2100; note that most of the results are presented only for the last time step) under two climate change scenarios on both extremes (i.e., low-emissions scenario SSP1–2.6 and high-emissions scenario SSP5–8.5) under the most recent Climate Model Intercomparison Project phase 6 (CMIP6) framework. We used spatially high-resolution (5 arc-min, or ∼10 km at the equator) data from eight global circulation models (GCMs), downscaled and bias corrected by WorldClim^35^ (experimental procedures; Figure S1). We were thus able to map how the HLZs would spatially change over this century.

Among the largest changes under the climate change scenarios assessed by 2081–2100 in HLZs is the shrinking of the boreal forest zone, from 18.0 million km^2^ (Mkm^2^) to 14.8 or 8.0 Mkm^2^ (SSP1–2.6 or SSP5–8.5, respectively). Under future conditions, the largest positive net increase is the growing tropical dry forest zone, from 15.0 to 19.2 or 27.7 Mkm^2^, ending up being globally the largest zone together with tropical desert (see Table S1). The largest reduction in relative terms occurs in the tundra (−39% or –75%; i.e., almost disappearing under SSP5–8.5 from 9.1 to less than 2.5 Mkm^2^) and boreal forest (−20% or –57%). In contrast, the largest increase in relative terms would occur in boreal desert (+159% or +75%), temperate desert (+24% or +110%), and temperate forest (+48% or +118%) (Table S1). Particularly alarming is the potential net increase in the combined area of “desert zones,” from 59.7 to 62.7 or 64.3 Mkm^2^ (of a total 150 Mkm^2^ included in the analysis), indicating drier conditions in many regions.

**Figure 2:**
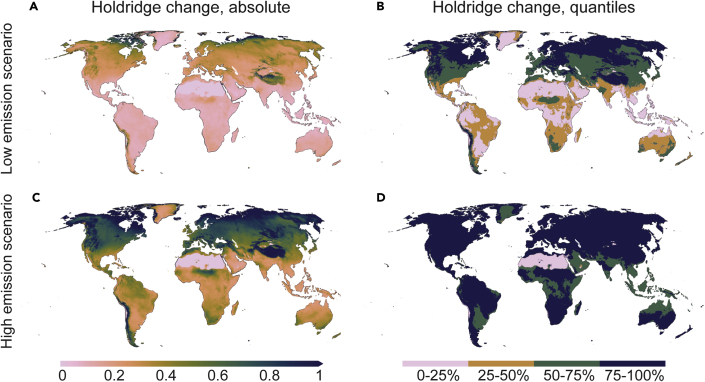
Holdridge zonal change under two climate change scenarios for 2081–2100 (A–D) Absolute change (A and C) and quantiles (B and D) of low-emissions scenario, SSP1–2.6 (A and B) and high-emissions scenario, SSP5–8.5 (C and D). The absolute change is scaled so that value 1 refers to the distance between two Holdridge zone centroids (Figures 1 and S6; see also experimental procedures), meaning a distance that is required to move from the “center” of one zone to another. Note that quantile limits were derived relative to SSP1–2.6 for both climate change scenarios; i.e., we used the SSP1–2.6 results to map the change thresholds for quantiles and used these same thresholds for SSP5–8.5 so that scenarios would be comparable. See direction of change in Figure S4.

As the Holdridge concept allows one to assess not only changes in climate zones, but also the magnitude and direction of change (experimental procedures; Figure S6), we were able to map these changes (Figures 2 and S4) even in areas where the climate zone itself would remain unchanged in future conditions. To measure this change, we assessed for each grid cell the distance between the future location and the baseline location within the HLZ triangle, as illustrated in Figure S6. The distance was normalized with the distance between two Holdridge zone centroids, so that a change of one unit refers to a change that would be required to move from the centroid of one zone to another. The largest change in both future scenarios (SSP1–2.6 and SSP5–8.5) occurs in the polar regions, the Sahel, and the major mountain areas (Figure 2). For both emission scenarios, the majority of the regions will develop toward more arid conditions, except for parts of northern Africa and the Middle East, where conditions would become wetter (Figure S4).

### Low resilience increases vulnerability to HLZ changes

Societies have varying abilities to react to changes in climatic zones, depending on their resilience^1^ to cope with the potential disruptions. Thus, we further linked the gridded global dataset of resilience^34^ with 5 arc-min resolution (∼10 km at the equator) for the year 2010 (experimental procedures) to the hotspot analysis to identify the most vulnerable areas. The low-resilience areas (bottom 25th percentile) cover a large part of South Asia, the Middle East, and Africa (Figure S5D).

**Figure 3:**
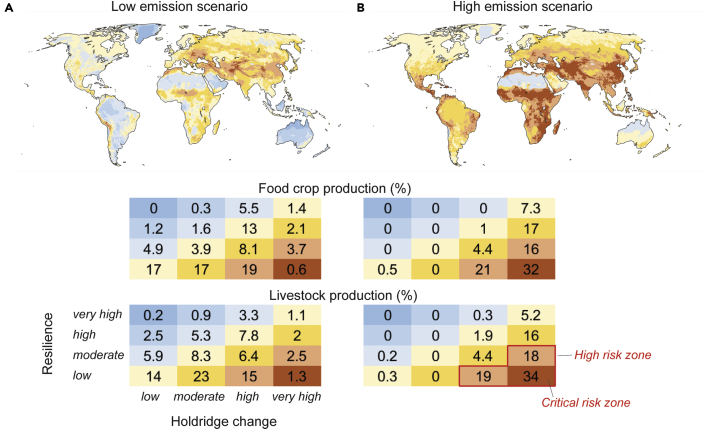
Classified Holdridge change and resilience as well as their relation to livestock and food crop production extent (A and B) Data are shown for the low-emissions scenario, SSP1–2.6 (A), and high-emissions scenario, SSP5–8.5 (B) for 2081–2100. The classes for Holdridge change and resilience are based on area-weighted quantiles: 0%–25% (low), 25%–50% (moderate), 50%–75% (high), 75%–100% (very high). High-risk zone is defined as where resilience is moderate and Holdridge change very high, or resilience is low and Holdridge change is high or very high. Similar to Figure 2, Holdridge change quantiles were always derived relative to the SSP1–2.6 scenario, i.e., we used the SSP1–2.6 results to map the change thresholds for quantiles and used these same thresholds for SSP5–8.5 so that the scenarios would be comparable. See Tables S2–S5 for tabulated results and Table S6 for sensitivity analysis of resilience percentile threshold.

When considering resilience with the HLZ change, the difference between the two scenarios is remarkable. Under the low-emissions scenario (SSP1–2.6), the areas under most critical risk (i.e., lowest 25th percentile of resilience and top 25th percentile of change in HLZ) lie in the Sahel and the Middle East, covering around 1% of global crop and livestock production (Figure 3A). If nations are not able to halt the growth in GHG emissions and the global community ends up following the path of the most extreme climate change scenario (SSP5–8.5), the portions may reach 32% for crop production and 34% for livestock (Figure 3). These most critical areas would then cover most of the Middle East, a large part of South Asia, and parts of sub-Saharan Africa and Central America (Figure 3B). Remarkably, over two-thirds of crop production and over 70% of global livestock production would be under high and critical risk zones (combination of high change in HLZ and low resilience or very high change in HLZ and high to moderate resilience, see Figure 3).

As the results are sensitive to the resilience percentile (25^th^ percentile) chosen for low-resilience class, we tested this sensitivity by doing the analyses with the 20^th^ to 30^th^ percentiles, too. We found that the crop and livestock production in the critical-risk zone under the high-emissions scenario would vary between 28% and 36% and between 30% and 39%, respectively (Table S6).

### Large proportion of food production beyond SCS

**Figure 4:**
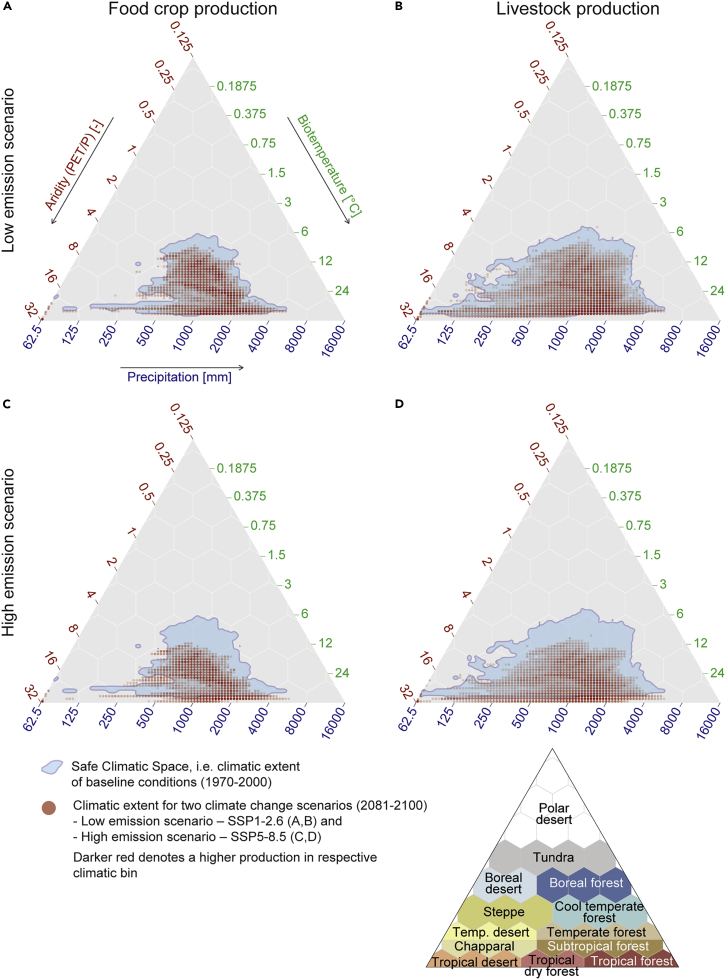
Safe climatic space and climatic extent of future climate change scenarios for food crop production and livestock production (A–D) SCS and future climatic extent are mapped to the Holdridge variables for the low-emissions scenario, SSP1–2.6 (A and B), and high-emissions scenario, SSP5–8.5 (C and D), for 2081–2100. Light blue denotes the SCS, i.e., the baseline climatic conditions in which 95% of the highest livestock and crop production areas are currently located (experimental procedures, Figure S3). The transparency of the red dots illustrates the amount (higher saturation means larger amount) of livestock and crop production under the future climatic conditions (similarly, 95% of global livestock and crop production included) in the respective climatological bin. “PET” stands for potential evapotranspiration and “P” stands for precipitation.

The estimated large shifts in climate zones (Figure 2) risk pushing remarkable parts of global food production outside the SCS. We first defined the SCSs separately for crop production and livestock production by mapping the baseline climatic conditions in which 95% of the highest crop and livestock production areas are located (experimental procedures, Figure S3). We then compared the future climatic conditions in each spatial location (5 arc-min grid) with these SCSs, separately for these two food production sectors, and were thus able to identify the areas at risk of falling outside the SCS (Figure 4).

Comparing the SCSs (i.e., climatic niche) for crop and livestock production areas (blue area in Figure 4; Figure S3), we can see that, as expected, the SCS is much larger for livestock. The SCS for livestock production spans over drier as well as wetter areas, compared with that for crop production, while the lower boundary for biotemperature is relatively similar for both (between 3°C and 6°C) (Figure 4).

**Figure 5:**
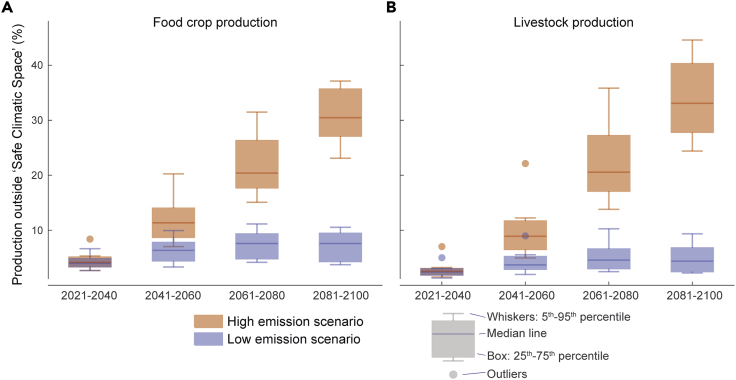
Temporal evolution of global food crop production and livestock production that would fall outside safe climatic space (A and B) The boxplots show the proportions of global crop production (A) and livestock production (B) that would fall outside the SCS across the eight global circulation models (GCMs; see experimental procedures) for the years 2021–2040, 2041–2060, 2061–2080, and 2081–2100. Results are shown for both low-emissions scenario (SSP1–2.6) and high-emissions scenario (SSP5–8.5). SCS refers to climatic conditions where the majority (95%) of livestock or food production exists within baseline conditions. Errors bars (i.e., whiskers) represent the 5^th^–95^th^ percentile range across GCMs.

Our results show strong contrasts between the two examined climate scenarios. In the low-emissions scenario (SSP1–2.6) only rather limited parts of current crop production (8%; 4%–10% with 5^th^–95^th^ percentile confidence interval across models; see Figures 4A and 5A and Table S7) and livestock production (5%; 2%–8%; Figures 4B and 5B) would fall outside the SCS. With the high-emissions scenario (SSP5–8.5), globally as much as 31% (25%–37%) of the crop production and 34% (26%–43%) of the livestock production would be at risk for facing conditions beyond the corresponding SCSs (Figures 4C, 4D, 5A, and 5B). When looking at the evolution over time, we found that the two emission scenarios used resulted in rather similar outcomes for the first two time steps (2021–2040, 2041–2060), after which there was a strong divergence between them (Figure 5).

**Figure 6:**
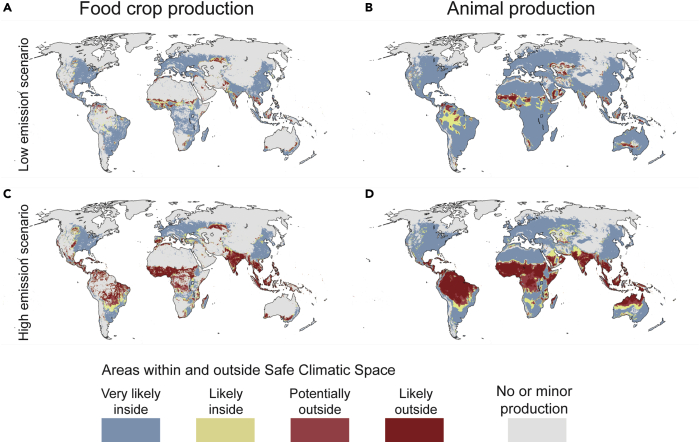
Extent of food crop production and livestock production that would fall within and outside safe climatic space (A–D) “No or minor production” refers to the remaining 5% of the respective areas. Results are presented separately for low-emissions scenario (SSP1–2.6) (A and B) and high-emissions scenario (SSP5–8.5) (C and D). The likelihood of crop production (A and C) and livestock production (B and D) falling outside the SCS was determined based on the number of global circulation models (eight in total) showing that the SCS is left: 0 (very likely inside), 1–3 (likely inside), 4–6 (potentially outside), 7–8 (likely outside). SCS refers to climatic conditions where the majority (95%) of livestock or food production exists within baseline conditions. See globally aggregated results in Table S7.

Further, the risks for individual countries appear very heterogeneous: in 52 of the 177 countries—a majority being European—the entire food production system would stay within the SCS (Figure 6; Data S1). This does not free those countries from experiencing changes in their climatic conditions (Figures 1A–1C), but the projected future climatic conditions are currently experienced elsewhere in the world and are thus not novel globally. In the worst position would be, e.g., Benin, Cambodia, Ghana, Guinea-Bissau, Guyana, and Suriname, where, alarmingly, over 95% of both crop and livestock production would move beyond the SCS.

Unfortunately, in many of the highly affected areas the resilience to cope with the change is currently low (Figure S7). Critical areas—both facing actual risk of falling outside the SCS and already low in resilience—can be found extensively in the Sahel region, the horn of Africa, and South and Southeast Asia (Figure S7). Particularly Benin and Cambodia (over 95% of food production beyond the SCS and under low resilience), as well as Burkina Faso, Chad, Côte d'Ivoire, Guinea-Bissau, Niger, and Sierra Leone (over 85%), would face severe challenges in producing their food if the world community fails to combat climate change and follows the high-end SSP5–8.5 scenario and their resilience remains low. Altogether, 20% of the world's current crop production and 18% of livestock production are at risk of falling outside the SCS with low resilience to cope with that change (Table S7; Data S1).

## Discussion

Our findings reinforce the existing research^17^^,^^36^^,^^37^ in suggesting that climate change forces humanity into a new era of reduced validity of past experiences and dramatically increased uncertainties. Whereas changes are expected in all climatic zones across the planet (Figure 1), we were able to detect crop and livestock production areas that would fall outside the SCS (Figure 6), as well as highlighting areas that are at highest risk due to their concurrent low resilience (Figure S7). The ability of individual countries to face these projected changes and their potential effects, such as environmental refugees^38^ and growing importance of international food trade in conditions where local food production cannot meet the demand,^39^ varies considerably.

We further highlight the drastic differences in the impacts on food production between low- and high-emissions scenarios, stressing the importance of limiting global warming to 1.5–2°C. These impacts of changes in climatic conditions on food production will likely be amplified by other factors, such as population growth,^40^ land degradation,^38^ and other environmental challenges related to sustainable food production,^41^ as well as increased risk of climate extremes.^42^^,^^43^ Alarmingly, the same areas where food production has the highest risk of falling beyond the SCS are projected to increase their population,^40^ and thus food demand, during this century. The predicted increase in desert areas (Table S1) will potentially also alter the local biogeochemical processes that are strongly controlled by water and temperature.^44^^,^^45^ In addition, an increasing asynchrony of the growing season and water availability will likely have additional effects on biodiversity and food production.^46^ These potential impacts illustrate soundly the multifaceted effects that greatly challenge global food production, quality of food, and food prices, among many other issues.^47^

Therefore, the scrutiny of these additional factors is crucial for future research, by building on our current analysis. This would, however, require tools and models that are beyond the scope of our approach. Further, many of these factors, such as future changes in climate variability and climate extremes, remain uncertain in GCMs^48^^,^^49^ and thus cannot yet be included in the analysis. Further, we acknowledge that using the 2010 data for spatial distribution of food production and of resilience limits the analysis of how future changes would influence the current production areas. While this does not take into account potential changes in the areas where food is produced or the impact of climate change on yields, it illustrates well the current production areas that might face an elevated risk under future conditions. Further, while the inclusion of scenarios of future food production impacts would be important, the high uncertainty of the future scenarios^11^ led us to leave those for forthcoming studies.

To conclude, future solutions should be concentrated on actions that would both mitigate climate change and increase resilience in food systems^50^, ^51^, ^52^ and societies,^34^ increase food production sustainability that respects key planetary boundaries,^41^ adapt to climate change by, for example, crop migration,^53^ and foster local livelihoods in the most critical areas. All this calls for global partnerships and solidarity, as well as innovative cross-sectoral thinking, to find the needed solutions. Our analyses should thus be linked to other sectors in future studies, first to better understand the cumulative pressure on different sectors in future scenarios and then to seek for future opportunities to secure sustainable development and equity.

## Experimental procedures

### Resource availability

#### Lead contact

Further information and requests for resources and reagents should be directed to and will be fulfilled by the lead contact, Matti Kummu (matti.kummu@aalto.fi).

#### Data and code availability

All input data used for the study are openly available, as stated in the article, and speficied in the github site linked below.

The code generated during this study is available at github: https://github.com/matheino/holdridge.

#### Materials availability

The tabular dataset generated during this study is provided as Data S1: “Country level results for food crop production and livestock production area outside safe climatic space. The key spatial datasets generated during this study are available at Zenodo: https://doi.org/10.5281/zenodo.4700860.

### Data

HLZ is an ensemble of 38 life zones that were merged here to 13 zones (following Leemans^30^ and further combining two tropical forest classes) (Figure 1D). HLZs are based on the following variables: annual precipitation, aridity indicator (ratio between average annual potential evapotranspiration [PET] and precipitation), and biotemperature (see maps in Figure S1) using data from WorldClim v.2.1, based on approximately 9,000 and 60,000 weather stations.^35^ HLZs are especially useful for assessing spatiotemporal and climatic changes locally. To estimate the current and future distribution of these zones, we calculated the parameters needed for determining the HLZ based on the open access WorldClim v.2.1 dataset,^35^ which provides monthly climate data averaged over the baseline period of 1970–2000 as well as future scenarios. We used data for these baseline climate conditions and future climate change predictions for four time steps: 2021–2040, 2041–2060, 2061–2080, and 2081–2100. All these were based on eight GCMs and two climate change scenarios at both extremes (i.e., low-emissions scenario SSP1–2.6 and high-emissions scenario SSP5–8.5) under the most recent CMIP6 framework. The GCMs included are as follows: BCC-CSM2-MR, CNRM-CM6-1, CNRM-ESM2-1, canesm5, IPSL-CM6A-LR, MIROC-ES2L, MIROC6, and MRI-ESM2-0.

All data were downloaded from WorldClim^35^ with 5 arc-min resolution (or ∼10 km at the equator). The data were downscaled and bias corrected by WorldClim^35^ (more information about the methods is available at https://www.worldclim.org/data/downscaling.html).

For assessing the potential impacts of climate change on food production, we used openly available global spatial datasets. For crop production, we used the total crop production data from SPAM,^32^ which include 27 major food crops altogether (we intentionally left out 15 non-food crops labeled as non-food crops in the SPAM data,^32^ including, for example, sugarcane and sugar beet), for the year 2010 with resolution of 5 arc-min.

For the distribution of livestock production, we used Gridded Livestock of the World (GLW3)^33^ data for the year 2010 with the original resolution of 5 arc-min. We combined the major types of livestock (cattle, sheep, goats, pigs, chickens, horses, buffalo) into animal units (AU) following Holecheck et al.^54^ and the FAO:^55^

- cattle, 1.0 AU
- sheep, 0.15 AU
- goats, 0.10 AU
- horses, 1.8 AU
- buffalo, 0.7 AU
- chickens, 0.01 AU
- pigs, 0.2 AU

To quantify the resilience of human societies to cope with the future changes, we used the recent resilience concept by Varis et al.^34^ The concept is based on a composite index approach for combining geospatially adaptive capacity and environmental pressure on a global scale for the years 1990–2015 (here year 2010 was used to be consistent with crop production and livestock production data), resulting in raster maps over the globe's land surface area with a 5 arc-min resolution.

### Methods for Holdridge life zone calculations

Annual precipitation (mm year^−1^) was calculated from monthly precipitation data, as defined by the HLZ method,^19^ directly available from the WorldClim v.2.1 dataset^35^ (Figure S1). Biotemperature was calculated based on the monthly average temperature. As the daily average temperature was not available for future scenarios, we estimated the monthly average temperature as the average of monthly minimum and maximum temperatures. The resulting bias was corrected using the mean, minimum, and maximum monthly temperatures of the baseline conditions. The months with mean temperature below 0°C were omitted from biotemperature calculations, as defined in the method.^19^ Note that, while in the original method^19^ months with temperatures over 30°C were omitted, we did not use this cap. We came to this solution by comparing the PET derived in Holdridge methods from biotemperature (see below, and Figure S2) and the satellite-observed PET [mm year^1^] and observing that the original PET method (Figure S2A) would not reflect well the observed PET (Figure S2F) in hot and dry areas, while the modified PET method, without the 30°C cap in biotemperature calculations, would result in much more reliable PET (Figure S2B). Once these modifications were done to the temperature datasets, the remaining monthly temperatures (°C) were averaged over a year. PET was estimated using the method described in Holdridge,^19^ i.e., by multiplying biotemperature by a constant value of 58.93. The aridity was calculated as PET ratio to mean total annual precipitation, and monthly PET values were summed over a year and then divided by annual precipitation (Figure S1). Finally, we used monthly minimum temperature data to map areas without any frost days (i.e., in all months, minimum daily temperature was above 0°C). These frost data were used to delineate temperate zones from sub-tropical ones (Figure 1D).

### Methods for estimating change in Holdridge life zones

Based on the data introduced above, we were able to define the HLZ for each 5 arc-min grid cell, for both current and future conditions (Figures 1A–1C; Table S1). We used the original method^20^ to define the life zone, as briefly explained below.

To implement the HLZ diagram computationally, we constructed a version in Cartesian coordinates from precipitation (*P* [mm]) and aridity (i.e. PET ratio to precipitation; *R* [-]) using the thresholds given by Holdridge.^19^ Bearing in mind that the HLZ diagram is an isosceles triangle and that its axes are logarithmic, and using the ranges of the *P* and *R* axes, a given value of *P* and *R* translates into Cartesian coordinates x and y (both with value range [0,1]) as follows:

*P′ = (log*_*2*_*(P) − log*_*2*_*(62.5 mm))/(log*_*2*_*(P) − log*_*2*_*(16,000 mm)) ∗ 1/mm,*

*R′ = (log*_*2*_*(R) − log*_*2*_*(0.125))/(log*_*2*_*(R) − log*_*2*_*(32)),*

*X = 0.5 ∗ (1 + P' − R'),*

*Y = 1 − P' − R'.*

Once we had the Cartesian coordinates for each grid cell, we were able to assign a Holdridge class to each cell. This was then used to estimate the change in future climate scenarios. To estimate the change, we used the ensemble median of the 8 GCMs (see above) and, instead of just mapping the cells where the HLZ class would change, we calculated the distance between the current and the future location (see Figure S6A) as well as the direction of change. With the distance, we were able to estimate the magnitude of the change in absolute terms, and when dividing that by mean distance between the two HLZ centroids we got the relative change. The direction of change, in turn, indicates whether the change is mainly due to higher biotemperature, wetter conditions, or larger PET ratio (see Figure S6B).

### Methods for spatial assessments

To extract spatial patterns for the changes in HLZs, for each raster cell, we scaled the change between current and future HLZ coordinates by dividing by the distance between two HLZ centroids. Hence, a change of one means that the observed change in the HLZ coordinates is equal to the difference between two HLZ centroids. The scaled HLZ change values were also divided into classes based on area-weighted percentiles: 0%–25% (low), 25%–50% (moderate), 50%–75% (high), and 75%–100% (very high).

To map the most critical areas with low capacity to cope with future changes, we used an indicator for resilience.^34^ For this purpose, the resilience data^34^ (Figure S5C), ranging between −1 and 1, was divided into area-weighted percentiles (Figure S5D), similar to the HLZ data.

After dividing the HLZ change and resilience values into the four percentile classes, we compared them with crop production in kilocalories^32^ (Figure S5A) and livestock production in animal units (see above) (Figure S5B). Namely, we analyzed how the extent of livestock and crop production relates to the changes in the HLZs and resilience. The analysis was conducted by summing the respective production data that fall into each of the HLZ change and resilience classes leading to 16 classes in total.

### Safe climatic space

We further assessed and estimated the crop and livestock production areas under risk of falling outside the corresponding SCS, i.e., moving beyond climatic conditions under which the majority (95%) of the food is currently produced under baseline conditions. To define and map the SCSs, we first placed each grid cell with, for example, food crop production in the Holdridge triangle (Figure 1D) using the baseline biotemperature, precipitation, and aridity climatic conditions. Once we had placed all the food crop production areas in the triangle, we got a cloud of the climatic conditions where food crops are currently produced. From this cloud of points, we filtered out the 5% smallest crop production areas, leaving the SCS area covering 95% of crop production (see Figure S3). Thus, the SCS is defined as the climatic space where 95% of crop production takes place. The calculations were conducted similarly for livestock production (Figure S3B).

Then we compared the future climatic conditions of these major production areas (also filtering out the smallest 5% for future conditions), and estimated which would fall beyond the SCS under both emission scenarios. Finally, utilizing simulation results across the eight GCMs, the likelihood of falling beyond the SCS was mapped for each grid cell, as well as being aggregated to the national level.

## supplemental information

Document S1. Figures S1–S7 and Tables S1–S7

Data S1. Country level results for food crop production and livestock production area outside safe climatic space

Document S2. Article plus supplemental information
