# Supplementary material for: Climate change risks pushing one-third of global food production outside the safe climatic space
Source: One Earth. 2021 May 21;4(5):720–9. doi: 10.1016/j.oneear.2021.04.017 (PMC8158176; doi:10.1016/j.oneear.2021.04.017)
Supplement: Document S1. Figures S1–S7 and Tables S1–S7 [file mmc1.pdf]

**One Earth, Volume *4***

## Supplemental information

# Climate change risks pushing one-third of global food production outside the safe climatic space

**Matti Kummu, Matias Heino, Maija Taka, Olli Varis, and Daniel Viviroli**

## Supplementary figures

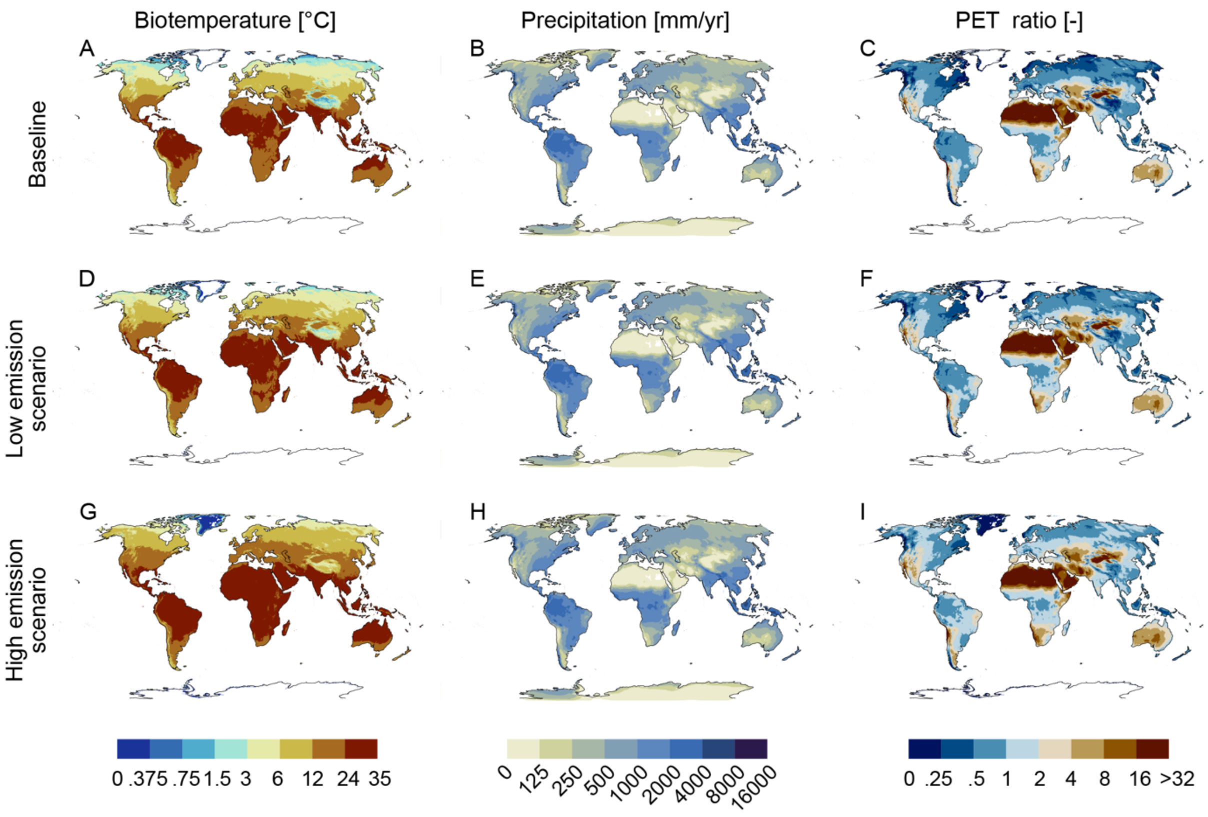


***Figure S1. The climatic variables used to estimate Holdridge Life Zones.*** *The climatic parameters include biotemperature (A, D, G), precipitation (B, E, H) and potential evapotranspiration ratio (C, F, I). Each mapped for baseline conditions 1970-2000 (A-C), for 2081-2100 under low emission scenario (SSP1-2.6) (D-F) and under high emission scenario (SSP5-8.5) (G-I). Data used to calculate these is from WorldClim v2.1 ref* [1]. *See Experimental Procedures for how the individual components were calculated.*

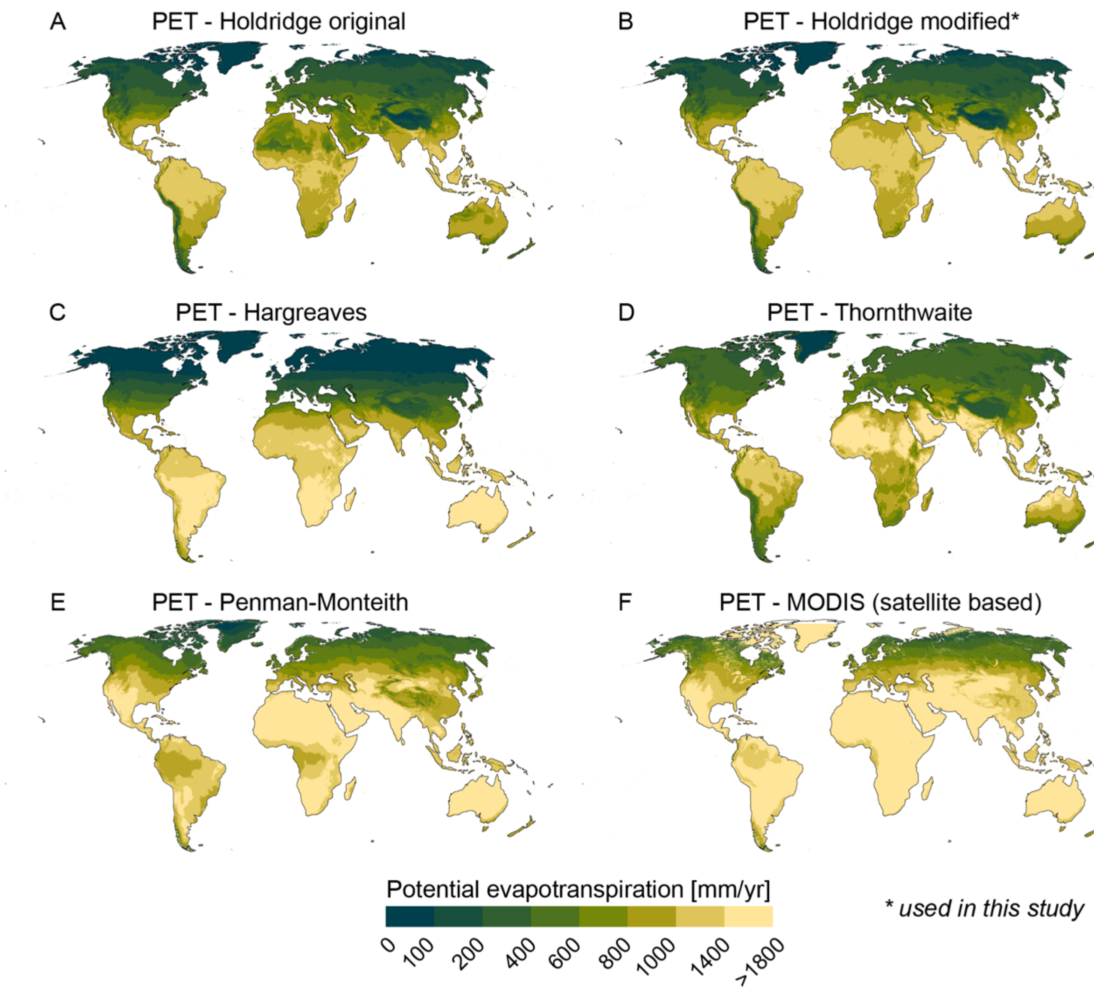


***Figure S2. Different methods to calculate the potential evapotranspiration (PET).*** *Original Holdridge method (A) can be compared to the modified method used here (see Experimental Procedures) (B), as well as other methods to estimate PET. Panels A-D were calculated as a part of this study, using data for years 1970-2000 from WorldClim v2.1 ref*[1]*. Data for PET using the Penman-Monteith method (E) is from Trabucco and Zomer*[2] *and satellite-based MODIS estimates (F) from NTSG*[3]*.*

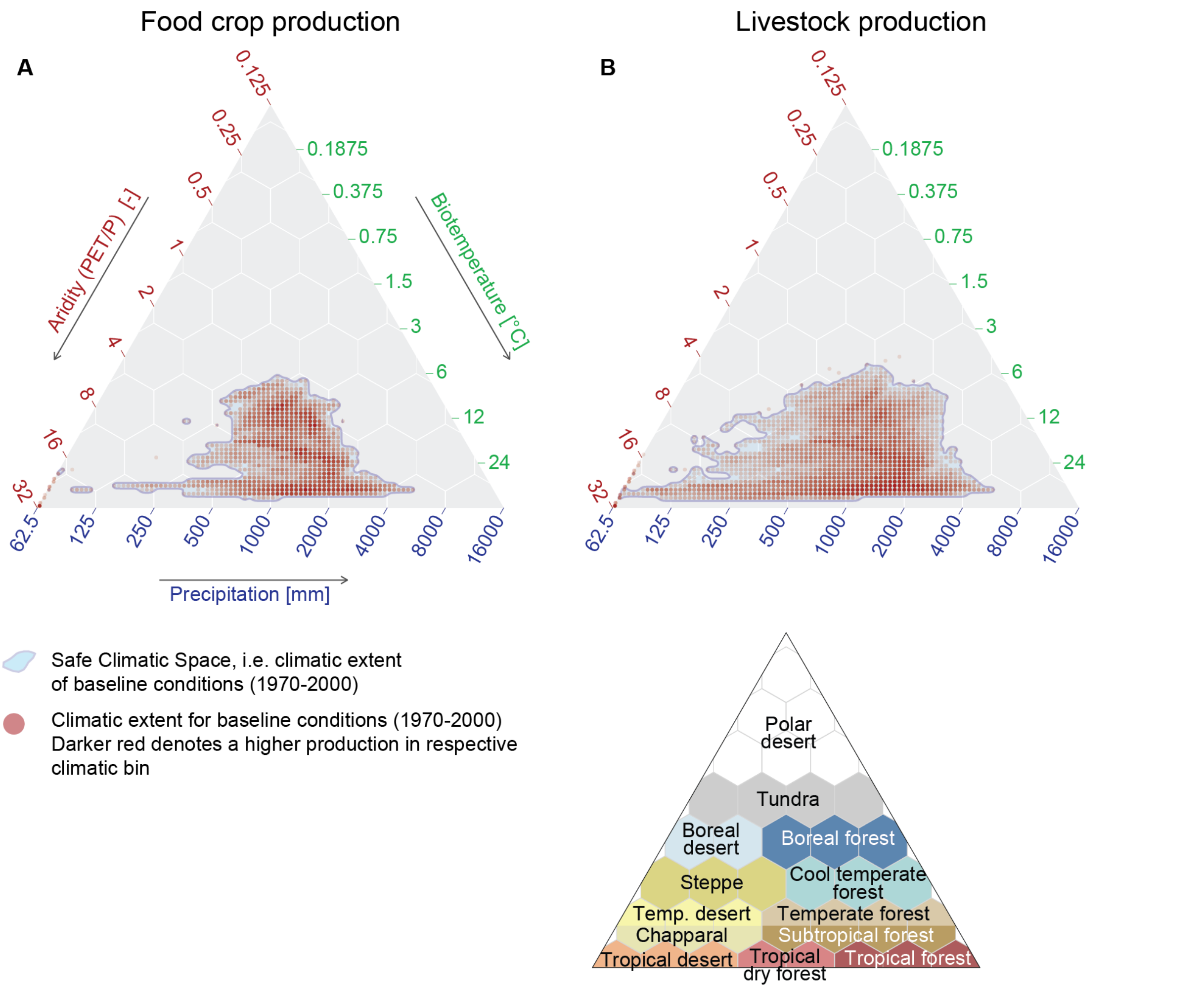


***Figure S3. Safe Climatic Space for food crop production and livestock production.*** *Food crop production (A) and livestock production (B) mapped to the Holdridge climatic variables for the baseline conditions 1970-2000. Light blue area denotes the 'Safe Climatic Space', i.e., the climatic conditions for these baseline conditions in which 95% of largest population and food production areas are located in (Experimental Procedures). The transparency of the red dots illustrates the amount (higher saturation means larger amount) production under the same baseline conditions (equally 95% of current global food production included) in the respective climatological bin. PET stands for potential evapotranspiration and P for precipitation.*

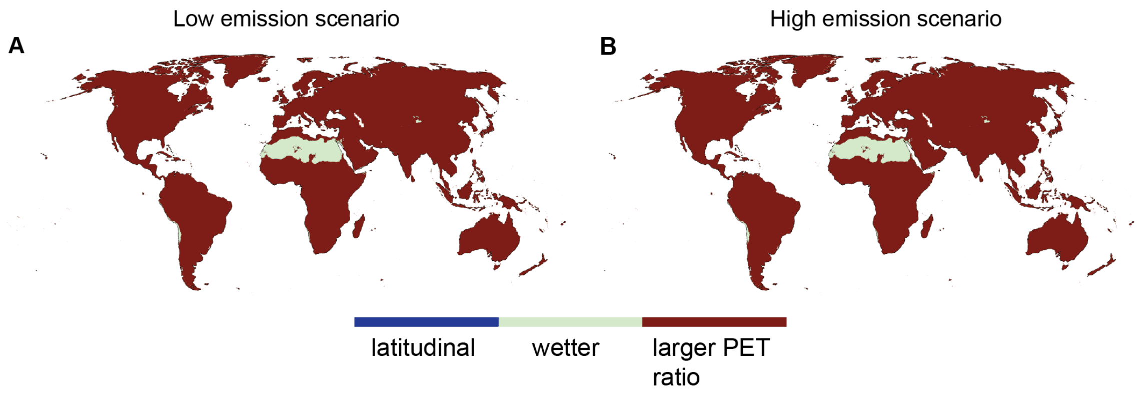


***Figure S4. Direction of change in Holdridge Life Zones.*** *Dominant component in Holdridge Life Zone change (see Experimental Procedures; Figure S6) for 2081-2100 under low emission scenario (SSP1-2.6) (A) and high emission scenario (SSP5-8.5) (B).*

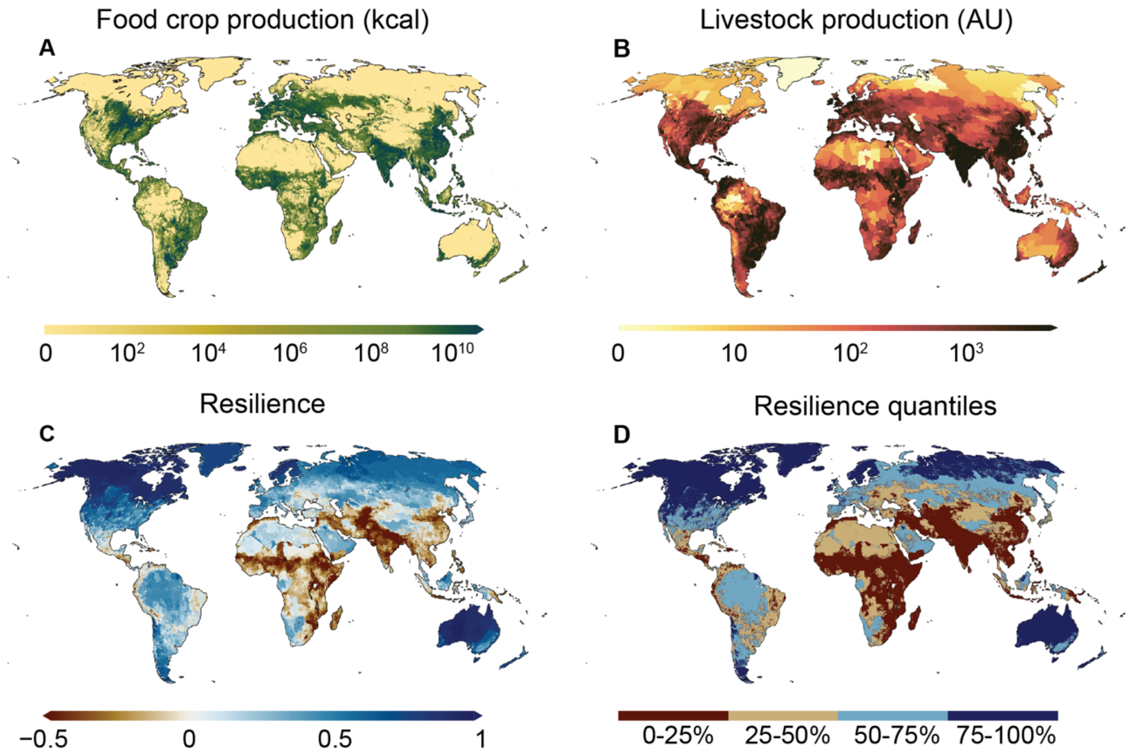


***Figure S5. Maps for additional input data used in the analysis.*** *The input data mapped for food crop production (A), livestock production (B), and resilience (C,D). Food crop production from SPAM*[4]*, livestock production from the Gridded Livestock of the World (GLW 3) database*[5] *and resilience from Varis et al* [6]*. AU stands for Animal Units (see Experimental Procedures).*

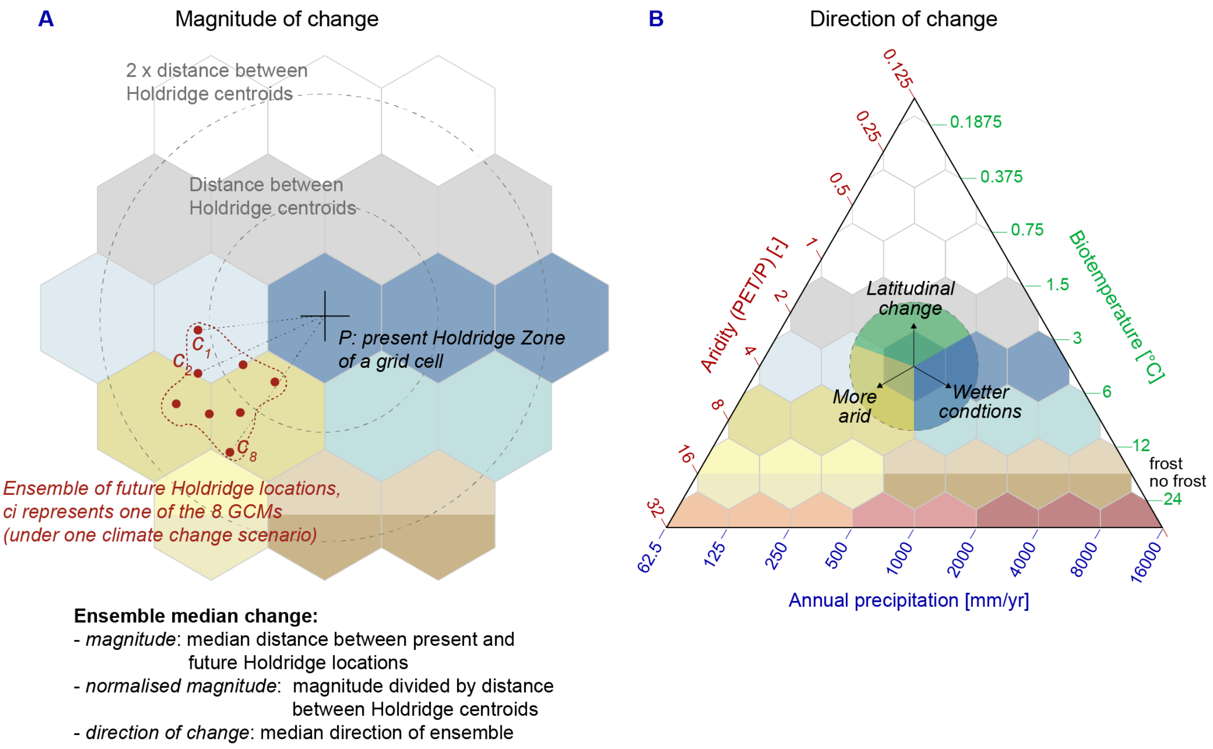


***Figure S6. Summary of the methods to calculate the ensemble median change.*** *Magnitude of change (A) and direction of change (B). Note: the actual distance and direction calculations were done using cartesian coordinates (see Experimental Procedures). PET stands for potential evapotranspiration and P for precipitation.*

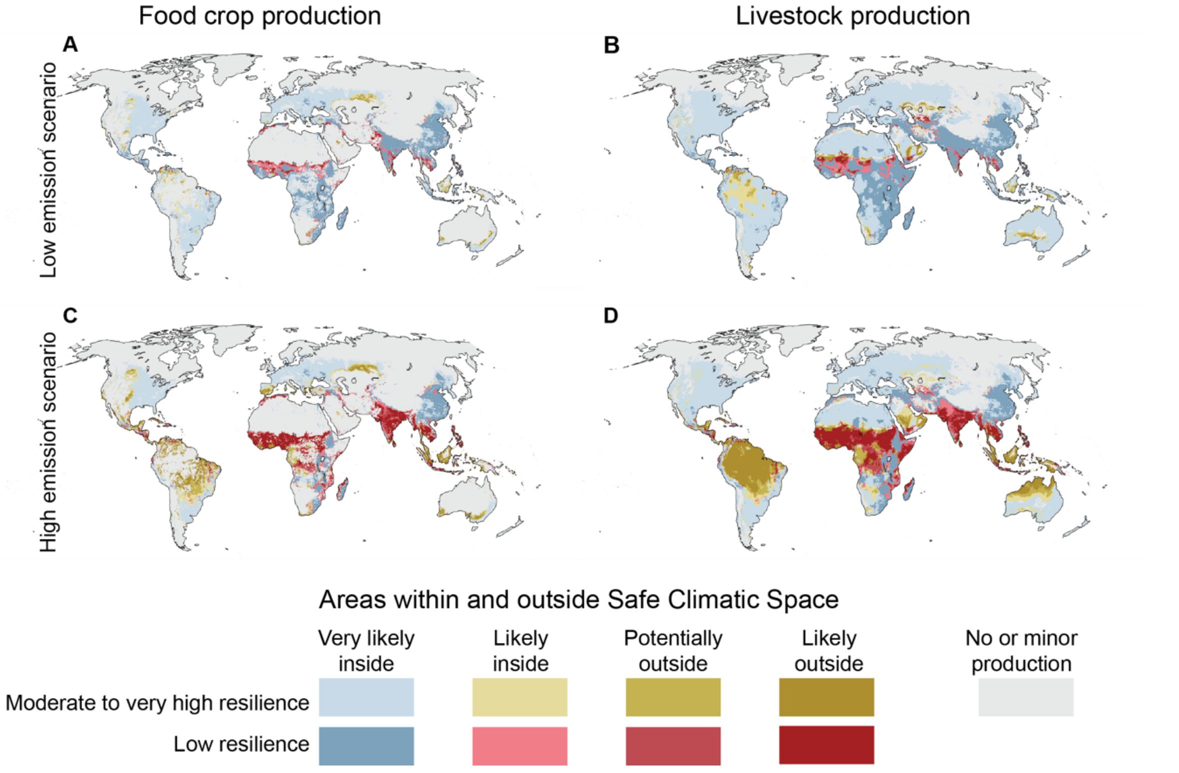


***Figure S7. Extent of food crop production and livestock production that would fall within and outside Safe Climatic Space (SCS).*** *'No or low production' areas refer to the remaining 5% of the respective areas. Low resilience refers to the bottom 25th percentile of resilience (see Figure S5D). Results are presented separately for low emission scenario (SSP1-2.6) (A, B) and high emission scenario (SSP5-8.5) (C, D). The likelihood categories of crop production (A, C) and livestock production (B, D) falling outside SCS were determined based on the number of Global Circulation Models (GCMs) (8 in total) showing that the SCS is left: 0 (very likely inside), 1-3 (likely inside), 4-6 (potentially outside), 7-8 (likely outside). SCS refers to climatic conditions where the majority (95%) of livestock or food production exist within baseline conditions. See globally aggregated results in Table S7.*

# Supplementary tables

***Table S1. Change in area of Holdridge Life Zones between baseline and future scenarios for 2081-2100.*** *Areas in 1000 $km^2$ of Holdridge zones on baseline (1970-2000) as well as future (2081-2100) conditions under low emission scenario (SSP1-2.6) and high emission scenario (SSP5-8.5).*

| Holdridge Zone | Baseline [1000 $km^2$] | Low emission scenario [1000 $km^2$] | High emission scenario [1000 $km^2$] |
|---|---|---|---|
| Polar Desert | 16,464 | 13,779 (–16.3%) | 12,370 (–24.9%) |
| Tundra | 9,131 | 5,572 (–39.0%) | 2,329 (–74.5%) |
| Boreal Desert | 911 | 2,354 (+158.5%) | 1,594 (+75.0%) |
| Boreal Forest | 18,513 | 14,760 (–20.3%) | 8,028 (–56.6%) |
| Steppe | 10,743 | 11,056 (+2.9%) | 9,259 (–13.8%) |
| Cool Temperate Forest | 12,301 | 14,957 (+21.6%) | 15,911 (+29.4%) |
| Temperate Desert | 3,819 | 4,719 (+23.6%) | 8,029 (+110.2%) |
| Temperate Forest | 3,368 | 4,984 (+48.0%) | 7,346 (+118.1%) |
| Chapparal | 11,018 | 9,270 (–15.9%) | 7,070 (–35.8%) |
| Subtropical Forest | 21,936 | 16,935 (–22.8%) | 13,471 (–38.6%) |
| Tropical Desert | 16,705 | 21,518 (+28.8%) | 26,027 (+55.8%) |
| Tropical Dry Forest | 15,036 | 19,213 (+27.8%) | 27,659 (+84.0%) |
| Tropical Forest | 10,588 | 11,414 (+7.8%) | 11,438 (+8.0%) |

***Table S2. Resilience and Holdridge change for food crop production under low emission scenario.*** *Food crop production ($10^{12}$ kcal) divided into resilience and Holdridge change quantiles under low emission scenario (SSP1-2.6). See map in Figure 3.*

| | Holdridge change | | | |
|---|---|---|---|---|
| **Resilience quantiles** | *0-25% [low]* | *25-50% [moderate]* | *50-75% [high]* | *75-100% [very high]* |
| 75-100% [*very high*] | 0.6 (0.006%) | 34 (0.3%) | 562 (5.5%) | 143 (1.4%) |
| 50-75% [*high*] | 121 (1.2%) | 167 (1.6%) | 1363 (13%) | 213 (2.1%) |
| 25-50% [*moderate*] | 499 (4.9%) | 401 (3.9%) | 827 (8.1%) | 373 (3.7%) |
| 0-25% [*low*] | 1756 (17%) | 1746 (17%) | 1925 (19%) | 62 (0.6%) |

***Table S3. Resilience and Holdridge change for food crop production under high emission scenario.*** *Food crop production ($10^{12}$ kcal) divided into resilience and Holdridge change quantiles under high emission scenario (SSP5-8.5). See map in Figure 3. Note: Holdridge change quantiles are derived from the SSP1-2.6 scenario.*

| | Holdridge change | | | |
|---|---|---|---|---|
| **Resilience quantiles** | *0-25% [low]* | *25-50% [moderate]* | *50-75% [high]* | *75-100% [very high]* |
| 75-100% [*very high*] | 0.02 (0.0002%) | 0.004 (0.00004%) | 1 (0.008%) | 739 (7.3%) |
| 50-75% [*high*] | 0.3 (0.003%) | 0.03 (0.0003%) | 101 (1.0%) | 1763 (17%) |
| 25-50% [*moderate*] | 4 (0.04%) | 0.4 (0.004%) | 446 (4.4%) | 1650 (16%) |
| 0-25% [*low*] | 48 (0.5%) | 2 (0.02%) | 2167 (21%) | 3272 (32%) |

***Table S4. Resilience and Holdridge change for livestock production under low emission scenario.*** *Livestock production ($10^6$ AU) divided into resilience and Holdridge change quantiles under low emission scenario (SSP1-2.6). See map in Figure 3. AU refers to Animal Units (Experimental Procedures).*

| | Holdridge change | | | |
|---|---|---|---|---|
| **Resilience quantiles** | *0-25% [low]* | *25-50% [moderate]* | *50-75% [high]* | *75-100% [very high]* |
| 75-100% [*very high*] | 3.8 (0.2%) | 22 (0.9%) | 79 (3.3%) | 25 (1.1%) |
| 50-75% [*high*] | 60 (2.5%) | 126 (5.3%) | 184 (7.8%) | 47 (2.0%) |
| 25-50% [*moderate*] | 138 (5.9%) | 194 (8.3%) | 150 (6.4%) | 59 (2.5%) |
| 0-25% [*low*] | 335 (14%) | 538 (23%) | 359 (15%) | 31 (1.3%) |

***Table S5. Resilience and Holdridge change for livestock production under high emission scenario.*** *Livestock production ($10^6$ AU) divided into resilience and Holdridge change quantiles under high emission scenario (SSP5-8.5). See map in Figure 3. Note: Holdridge change quantiles are derived from the SSP1-2.6 scenario. AU refers to Animal Units (Experimental Procedures).*

| | Holdridge change | | | |
|---|---|---|---|---|
| **Resilience quantiles** | *0-25% [low]* | *25-50% [moderate]* | *50-75% [high]* | *75-100% [very high]* |
| 75-100% [*very high*] | 0.1 (0.005%) | 0.05 (0.002%) | 6.8 (0.3%) | 123 (5.2%) |
| 50-75% [*high*] | 0.4 (0.01%) | 0.08 (0.003%) | 45 (1.9%) | 370 (16%) |
| 25-50% [*moderate*] | 5.8 (0.2%) | 0.2 (0.01%) | 104 (4.4%) | 431 (18%) |
| 0-25% [*low*] | 6.0 (0.3%) | 0.3 (0.01%) | 447 (19%) | 810 (34%) |

***Table S6. Results for sensitivity analysis of resilience threshold.*** *Sensitivity of the results to the impact of change in low resilience threshold on % of production falling to high change in Holdridge zone and low resilience class.*

| | Low resilience threshold | % of production falling to high change in Holdridge zone and low resilience class SSP1-2.6 | SSP5-8.5 |
|---|---|---|---|
| Food crop production | 20% | 0.3 % | 27.5 % |
| | 25% | 0.6 % | 32.1 % |
| | 30% | 1.2 % | 35.8 % |
| Livestock production | 20% | 1.0 % | 29.7 % |
| | 25% | 1.3 % | 34.5 % |
| | 30% | 1.8 % | 38.9 % |

***Table S7. Percentage of population and food production that would fall within and outside 'Safe Climatic Space' (SCS).*** *Low resilience refers to the bottom 25th percentile of resilience (see Figure S5D). Results are presented separately for low emission scenario (SSP1-2.6) and high emission scenario (SSP5-8.5). The likelihood categories of population and food crop production falling outside SCS were determined based on the amount of Global Circulation Models (GCMs) (8 in total) showing that the SCS is left: 0 (very likely inside), 1-3 (likely inside), 4-6 (potentially outside), 7-8 (likely outside). SCS refers to climatic conditions where the majority (95%) of livestock or food production exist within baseline conditions. See maps in Figure 6 and Figure S7.*

| | Ensemble median | Results based on 8 Global Circulation Models | | | |
|---|---|---|---|---|---|
| **Food crop production SSP1-2.6** | Outside SCS | Very likely inside | Likely inside | Potentially outside | Likely outside |
| Moderate to high resilience | 1.5% | 42.8% | 2.0% | 1.2% | 0.4% |
| Low resilience | 6.0% | 42.5% | 4.6% | 4.7% | 1.7% |
| **Total** | **7.6%** | **85.3%** | **6.7%** | **5.9%** | **2.2%** |
| **Food crop production SSP5-8.5** | | | | | |
| Moderate to high resilience | 6.4% | 35.1% | 4.5% | 2.9% | 4.1% |
| Low resilience | 24.8% | 22.1% | 5.2% | 6.6% | 19.6% |
| **Total** | **31.1%** | **57.2%** | **9.7%** | **9.5%** | **23.6%** |
| **Livestock production SSP1-2.6** | | | | | |
| Moderate to high resilience | 1.4% | 42.0% | 2.2% | 1.0% | 0.5% |
| Low resilience | 3.2% | 46.6% | 4.3% | 2.2% | 1.1% |
| **Total** | **4.6%** | **88.6%** | **6.5%** | **3.2%** | **1.7%** |
| **Livestock production SSP5-8.5** | | | | | |
| Moderate to high resilience | 10.1% | 31.5% | 3.4% | 3.3% | 7.6% |
| Low resilience | 23.7% | 21.7% | 7.0% | 7.3% | 18.3% |
| **Total** | **33.8%** | **53.2%** | **10.4%** | **10.6%** | **25.8%** |

## Supplemental references


1. Fick, S.E., and Hijmans, R.J. (2017). WorldClim 2: new 1-km spatial resolution climate surfaces for global land areas. International Journal of Climatology *37*, 4302–4315.

2. Trabucco, A., and Zomer, R. (2019). Global Aridity Index and Potential Evapotranspiration (ET0) Climate Database v2. figshare. Fileset. https://doi.org/10.6084/m9.figshare.7504448.v3.

3. NTSG (2020). MODIS Global Evapotranspiration Project (MOD16) (University of Montana, Numerical Terradynamic Simulation Group (NTSG)).

4. Yu, Q., You, L., Wood-Sichra, U., Ru, Y., Joglekar, A.K.B., Fritz, S., Xiong, W., Lu, M., Wu, W., and Yang, P. (2020). A cultivated planet in 2010 – Part 2: The global gridded agricultural-production maps. Earth System Science Data *12*, 3545–3572.

5. Gilbert, M., Nicolas, G., Cinardi, G., Van Boeckel, T.P., Vanwambeke, S.O., Wint, G.R.W., and Robinson, T.P. (2018). Global distribution data for cattle, buffaloes, horses, sheep, goats, pigs, chickens and ducks in 2010. Scientific Data *5*, 180227.

6. Varis, O., Taka, M., and Kummu, M. (2019). The Planet's Stressed River Basins: Too Much Pressure or Too Little Adaptive Capacity? Earth's Future *7*, 1118–1135.
